# Adhesive, Biocompatible, Antibacterial, and Degradable Collagen-Based Conductive Hydrogel as Strain Sensor for Human Motion Monitoring

**DOI:** 10.3390/molecules29235728

**Published:** 2024-12-04

**Authors:** Lixia Liao, Jiyuan Zhang, Jiaqi Ding, Chengzhi Xu, Lian Zhu, Yuanjing Hou, Sheng Li, Juntao Zhang, Benmei Wei, Haibo Wang

**Affiliations:** 1 School of Chemistry and Environmental Engineering, Wuhan Polytechnic University, Wuhan 430023, China; lxliao1024@126.com (L.L.); era0831@163.com (J.Z.); 18827971593@163.com (J.D.); xuchengzhi@whpu.edu.cn (C.X.); yljzl@whpu.edu.cn (L.Z.); houyuanjing@whpu.edu.cn (Y.H.); lfrcs@163.com (S.L.); zhangjt@whpu.edu.cn (J.Z.); benmeiwei@whpu.edu.cn (B.W.); 2 Hubei Key Laboratory of Quality Control of Characteristic Fruits and Vegetables, College of Life Science and Technology, Hubei Engineering University, Xiaogan 432000, China

**Keywords:** biocompatibility, collagen, strain sensor, adhesion

## Abstract

The conductive hydrogels (CHs) are promising for developing flexible energy storage devices, flexible sensors, and electronic skin due to the unique features of excellent flexibility and high conductivity. However, poor biocompatibility and antibacterial properties seriously limit their application in the biomedical field. Collagen, one of the main components of the extracellular Matrix (ECM), is the ideal matrix for constructing hydrogels due to good biocompatibility with human tissue. Here, dopamine–polypyrrole–collagen (DA-PPY-COL) hydrogel was constructed by dopamine-mediated pyrrole in situ polymerization in a collagen matrix. As a strain sensor, it can be affixed to different parts of the human body to monitor large-scale motion movements and fine micro-expressions in real time. The performance was attributed to its good self-adhesion, flexibility, and electrical conductivity. Biological experiments have shown that it has good antimicrobial properties, biocompatibility, and degradability, allowing the hydrogel to safely monitor human motor behavior. This work not only offers a material preparation strategy for constructing biomimetic electronic skin and wearable sensors but also demonstrates the great potential prospect for implantable degradable medical device applications.

## 1. Introduction

Recently, flexible materials have been widely considered for their excellent flexibility, softness, and ductility, which are applied in the field of sensing, such as pressure sensors [1], temperature sensors [2], thermal sensors [3], etc. Among them, strain sensors can detect real-time signals from joints, epidermis, and the heart for analysis and diagnostics [4,5,6]. When used for health monitoring, it is necessary to seek a high level of comfort and safety due to prolonged contact with the skin or tissue in addition to stable and sensitive signal output, which requires excellent adhesive, biocompatible, and antibacterial properties. Meanwhile, it also opens up the possibility of implantable medical applications.

Conductivity polymer hydrogels are a class of flexible materials with stable three-dimensional porous networks. It generally consists of a hydrogel flexible matrix and conductive active materials. Natural macromolecules with various origins, such as sodium alginate [7], chitosan [8], hyaluronic acid [9], cellulose [10], starch [11], etc., are often used as hydrogel matrices, especially for collagen from the natural extracellular matrix (ECM) [12,13,14]. It is considered an ideal raw material for constructing the biomimetic e-skin and flexible sensors due to its excellent biodegradability, bioactivity, and biocompatibility [14,15]. However, the stability of pure collagen-based hydrogels obtained through self-assembly is often poor, exhibiting fragile and brittle qualities. Furthermore, the inherent weak conductivity of pure collagen hinders the application of the strain sensor’s electronic skin. Remarkably, collagen is a protein consisting of three peptide chains twisted together, and it contains active groups, such as -NH_2_, -COOH, and a large amount of -OH, enhancing the interaction with the other molecules by amide bond, ester bond, Schiff base bond, coordination bond, etc. [16,17,18]. Constructing a double(multi)-network framework or modifying collagen with other micro/macromolecules are effective ways to enhance the mechanical properties of gel networks [6,19]. Gu et al. [6] constructed a highly stretchable, self-healing collagen-based CH via the metal coordination bonds between Al^3+^ and polyacrylic acid chains, the dynamic Schiff base bonds of collagen and dialdehyde carboxymethyl cellulose, hydrogen bond, and electrostatic interaction. The corresponding hydrogel strain sensor demonstrated accurate signal monitoring of human movements and a stable strain-detecting capability after being placed for a long time or in a cold environment. Subsequently, Gu and his research team [20] fabricated a double-network organhydrogel by combining the two networks—one created by the coordination reaction of polyacrylic acid with Zr^4+^ ions, and the other by the reaction of oxide hyaluronic acid with collagen. The obtained collagen-based hydrogel displayed high strength, self-healing, self-adhesion, biocompatibility, and excellent conductivity due to the introduction of several dynamic cross-linking mechanisms, such as Schiff base bond, collagen self-assembly, metal coordination, and hydrogen bond, and it can be utilized as a bioelectric and self-powered sensor to complete motion and physiological monitoring.

Furthermore, the introduction of conductive polymers into the hydrogel matrix can enhance the mechanical properties of gel networks, as well as the conductivity of hydrogels. Such as polyethylene dioxythiophene (PEDOT) [21], poly(2-ethylaniline) (PEAn) [22], poly (propynyl benzo thiazolone) (PTH) [23], polythiophene (PTh) [24], and polyaniline (PANI) [25]. The free lone-pair electrons can provide electrical paths for the mobile charge carriers, thus facilitating the regulation of conductivity. Polypyrrole (PPY) is a conducting polymer that is widely utilized because of its many exceptional properties, including its affordability, stability, high electroconductivity, environmental friendliness, biocompatibility, biodegradability, and nontoxicity. Regrettably, PPY tends to self-aggregate, and it is not uniformly distributed in the hydrogels. Resultantly, a continuous conductive network cannot be constructed and the mechanical properties of the hydrogels formed are also weak. Intriguingly, the PPY formed under dopamine-mediated action is then uniformly dispersed into the gel matrix, which is due to the enhanced hydrophilicity induced by DA and the stronger hydrogen bonding of PPY and gelatin methacrylate (GelMA)-polyacrylamide (PAM) double-network hydrogel matrix [26].

In this paper, a collagen-based strain sensor for human motion detection was prepared using collagen as the substrate and dopamine-modified polypyrrole as a conductive filler. The strain sensor can accurately and rapidly obtain human posture and physiological information. Furthermore, it has high dispersibility, remarkable antibacterial properties, excellent sensitivity, self-adhesion, flexibility, and degradability, which can greatly improve the safety and comfort of wearable strain sensors, thus providing the possibility for future applications in electronic skin and wearable sensors.

## 2. Results and Discussion

### 2.1. Structural Characterization of Hydrogels

**Scheme 1:**
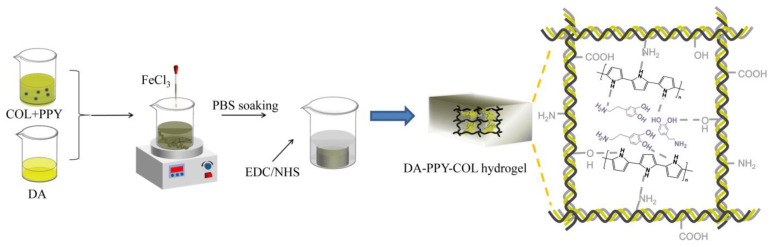
Preparation illustration of DA-PPY-COL composite hydrogels.

Scheme 1 demonstrates the construction of the DA-PPY-COL hydrogel. Firstly, the pyrrole monomers were thoroughly mixed with dopamine small molecules and a collagen solution, ensuring the uniform distribution of the pyrrole monomer within the collagen system. Subsequently, a solution of FeCl_3_ was slowly added dropwise to the system as a catalyst and stirred constantly at 4 °C to facilitate the complete oxidation of the pyrrole. This process promoted the formation of additional hydrogen bonds between PPY and COL. Meanwhile, the incorporation of dopamine, a dopant, strengthens the intermolecular noncovalent forces between the N-H bonds of the pyrrole ring and the -NH_2_ and -OH groups of the collagen polymer chains. Resultantly, pyrrole monomers were absorbed into the collagen hydrogel backbone. Then, the emulsion was gelatinized by soaking in a PBS solution. Finally, cross-linking agents 1-ethyl-3-(3-dimethylamino-propyl) carbodiimide (EDC) and N-Hydroxysuccinimide (NHS) were applied to rein-force the gel network, and the target product was obtained.

To ascertain the form in which the composite material exists, the viscoelastic of the DA-PPY-COL material was characterized by the oscillatory rheology test, which was conducted in the sweep frequency range from 0.1 to 10 Hz (Figure S1). Energy storage modulus (*G′*) values for all hydrogels were substantially greater than corresponding loss modulus values (*G″*) over the frequency range, suggesting that hydrogels with stable elastic networks were formed. Especially for the hydrogels containing 6% PPY and 8% PPY, their *G′* values were stable and higher than those of the other hydrogels in the whole frequency range, demonstrating that a suitable amount of rigid polypyrrole filled in the collagen matrix can maintain a good interaction with collagen, which plays a positive role in improving the mechanical properties of hydrogels.

**Figure 1:**
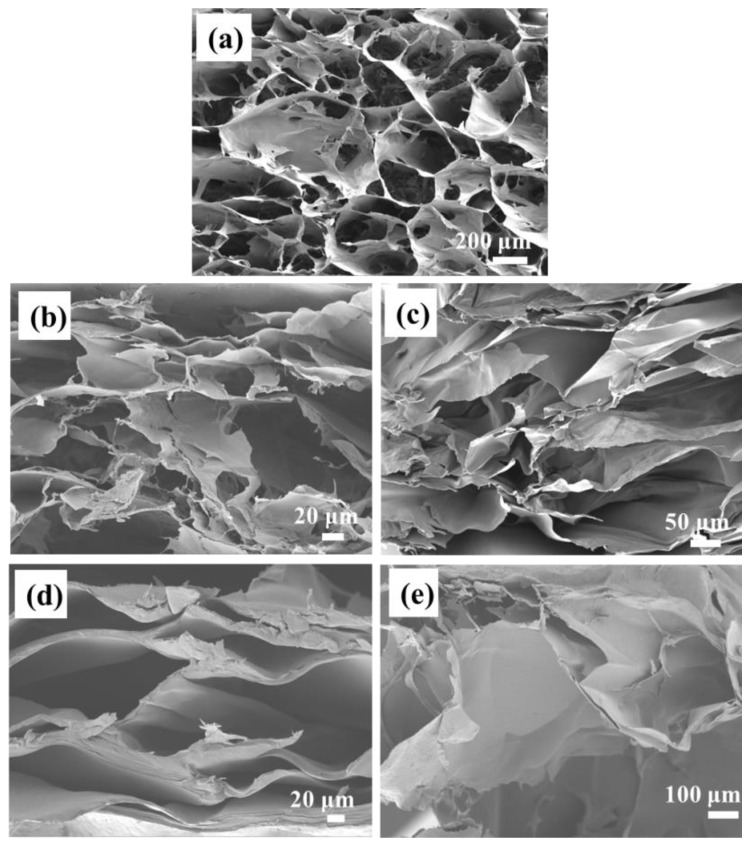
SEM images of the COL (**a**), DA-PPY-COL-4% (**b**), DA-PPY-COL-6% (**c**), DA-PPY-COL-8% (**d**), and DA-PPY-COL-10% (**e**).

Figure 1 displays the structural morphology of the hydrogel material. It exhibited a continuous, interconnected three-dimensional porous network structure, with a thin and locally compromised pore wall. After in situ polymerization of pyrrole, the pores of the formed hydrogels were significantly reduced, and the pore wall folds showed different morphologies with the addition of different amounts of pyrrole. Upon the polymerization of 6% pyrrole, the gel pore wall folds exhibited a uniform distribution within a continuous three-dimensional porous gel network, displaying a completely thin and brittle morphology. When the amount of pyrrole increased to 8%, the folds became significantly thicker, while the three-dimensional network structure remained intact and sturdy. As further increase to 10%, the pore wall folds gradually peeled off into layers, resulting in a partial transformation of the three-dimensional network porous structure into layers.

**Figure 2:**
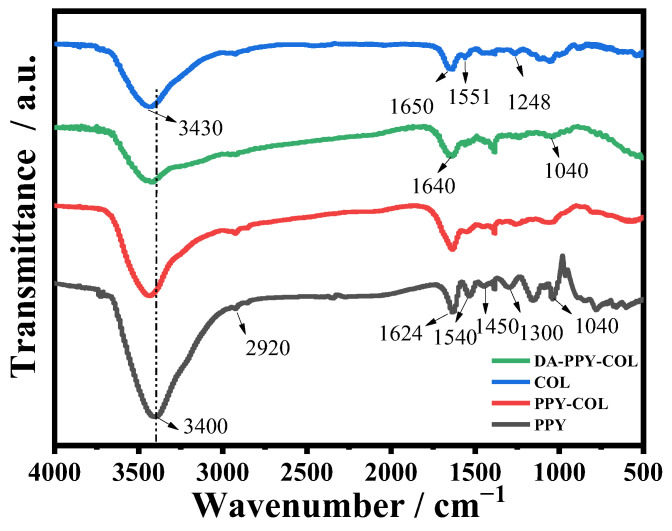
FTIR spectra of PPY, PPY-COL, COL, and DA-PPY-COL.

The FTIR spectra of PPY, PPY-COL, COL, and DA-PPY-COL hydrogels are shown in Figure 2. A prominent peak at 3400 cm^−1^ and a tiny peak at 2920 cm^−1^ of pure PPY were assigned to the N-H stretching vibration in the pyrrole ring [27]. Furthermore, several absorption peaks for the pyrrole ring at 1540, 1450, 1300, 1150, and 1040 cm^−1^, corresponding to C=C symmetric ring stretching, =C-N asymmetric ring stretching characteristic peaks, the C-C stretching, C-N the stretching vibration, and C-H in-plane vibration were also observed, respectively [4,28]. Some characteristic peaks were also present in the FTIR spectra of PPY-Col. However, some are different from those of pure PPY, which is due to the interaction between collagen macro-molecular chains and polypyrrole. For DA-PPY-Col hydrogel, it demonstrated similar FTIR spectra. Noteworthily, the distinctive peaks at 1640 cm^−1^ and 1040 cm^−1^ become broader, which could be the result of the superposition of dopamine characteristic peaks [29]. Furthermore, the FTIR spectra of COL hydrogel show its distinctive peaks, such as the -OH stretching vibration peak at 3430 cm^−1^, and the peaks at 1650 cm^−1^, 1560 cm^−1^, and 1260 cm^−1^, responding to amide I, II, and III bands [6]. These indicated the successful growth of surface dopamine-modified polypyrrole in COL hydrogel.

### 2.2. Adhesion Property of the DA-PPY-COL Hydrogel

**Figure 3:**
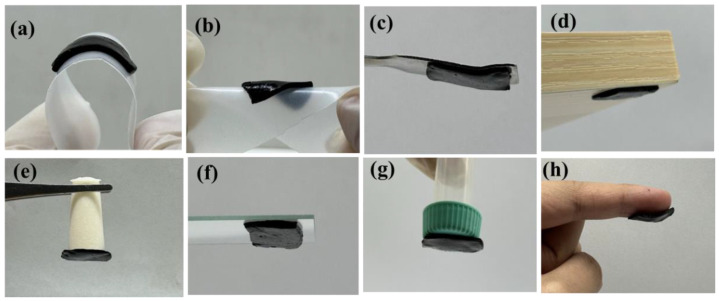
The adapting process of bending (**a**) and distorting (**b**) of DA-PPY-COL hydrogel. Adhesion property assessment of the DA-PPY-COL hydrogel. The DA-PPY-COL hydrogel can firmly stick to metal (**c**), wood (**d**), rubber (**e**), glass (**f**), plastic (**g**), and skin surface (**h**).

Good flexibility and adhesion are important properties of wearable stress sensors. They can ensure firm contact with the working interface, especially for large-scale movements, and resultantly guarantee steady and accurate conversion of mechanical signals into electrical impulses. As shown in Figure 3, CHs can be bent and twisted to a certain extent. Meanwhile, it also can be firmly attached to the surface of metal, wood, rubber, glass, plastic, and skin, exhibiting excellent viscosity.

The possible adhesion mechanism of DA-PPY-COL hydrogel is mainly attributed to DA, which has many catechol groups with universal adhesion through covalent or noncovalent bonds [30]. Moreover, DA provides strong hydrogen bonds between catechol groups and amino or carboxyl groups of proteins in the substrates. Thus, it is expected to be applied to human health monitoring equipment as electronic adhesives.

### 2.3. Biocompatibility of DA-PPY-COL Hydrogel

Biocompatibility represents a crucial parameter for assessing its potential as a stress-sensing application. Human venous endothelial cells (HUVEC) were used to perform living/dead staining after co-culturing with the hydrogels for 24 h. As shown in Figure S2a1–d3, large numbers of live cells (green fluorescence) and a small number of dead cells (red fluorescence) were detected, implying COL, COL-PPY, and DA-PPY-COL hydrogels are non-cytotoxic to HUVEC. Moreover, the CCK-8 method is a typical method for evaluating the cytotoxicity. The viability of HUVEC is shown in Figure S3. The viability of the cells was found to remain high after 24 and 48 h of coexistence of the gel extract with the cells. It indicates that the formation of PPY does not cause toxicity to the cells and the continuous PPY conductive network mediated by DA does not negatively affect the biocompatibility of the hydrogels. These demonstrate the high biocompatibility and predicted safety of the DA-PPY-COL hydrogels for human motion monitoring.

### 2.4. Antibacterial Properties of Collagen-Based Hydrogels

**Figure 4:**
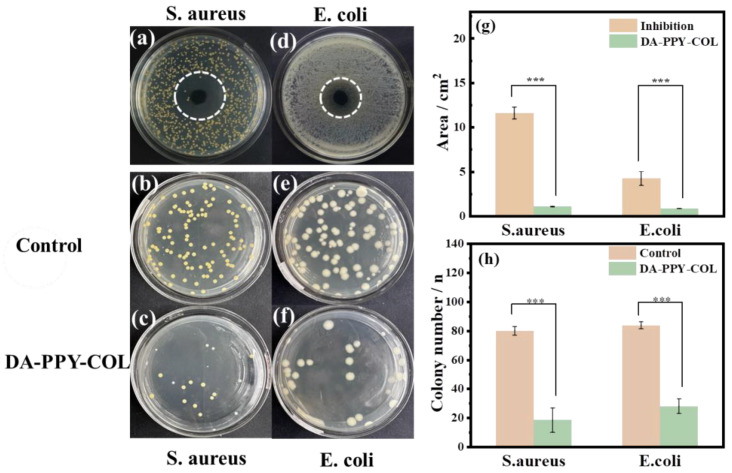
Antibacterial property test. Inhibition zone photographs of DA-PPY-COL hydrogels against *S. aureus* (**a**) and *E. coli* (**d**) and the areas of inhibition circles of *S. aureus* and *E. coli* (**g**), photographs of *S. aureus* (**b**) and *E. coli* (**e**) colonies of control group and photographs of *S. aureus* (**c**) and *E. coli* (**f**) colonies of test group containing DA-PPY-COL hydrogel on agar plates, and the responding statistic of colony number (**h**) against *E. coli* and *S. aureus*. *** *p* < 0.001, *n* = 3.

The required wearable strain sensors were designed to acquire electrical signals through direct contact with the skin. Nevertheless, prolonged skin contact may result in bacterial infections, which can cause discomfort and pose potential health risks. This may result in a higher frequency of hydrogel replacement and a shorter lifespan for devices that rely on hydrogel. Therefore, it is necessary to evaluate antibacterial performance. Antibacterial effect of DA-PPY-COL hydrogels against common bacterias, *Escherichia coli* (*E. coli*), a Gram-negative bacterium, and *Staphylococcus aureus* (*S. aureus*), a Gram-positive bacterium was investigated. As shown in Figure 4a,d, the antibacterial ring appeared on the agar plate for the DA-PPY-COL hydrogel, and a notable discrepancy was observed between the area of the antimicrobial ring and that of the hydrogel (Figure 4g).

Similar results are found in the spread agar plate method, which was used to directly observe the growth of bacterial colonies. The number of bacterial colonies on Agar plates treated with DA-PPY-COL hydrogel was significantly reduced in comparison with the control, suggesting their good antibacterial activity of DA-PPY-COL hydrogel. This is due to the fact that the interactions of positive charges on the PPY chains with the bacterial cell walls [31,32]. In addition, the rough, porous networks that formed inside the hydrogel due to the filling of polypyrrole also significantly influenced bacterial interactions, which improved antibacterial activity [33]. Furthermore, DA-PPY-COL hydrogels showed better inhibition against *S. aureus* than *E. coli* because the monolayer of *S. aureus* is composed of a phospholipid called lysine phosphatidylglycerol (Lys-PG), whose positive charge makes the *S. aureus* membrane resistant to PPY materials [34]. The excellent antibacterial properties of DA-PPY-COL hydrogels can ensure safe sensing detection when it is directly attached to skin and tissue, showing the potential for use in implantable medical devices.

### 2.5. Biodegradable Properties

**Figure 5:**
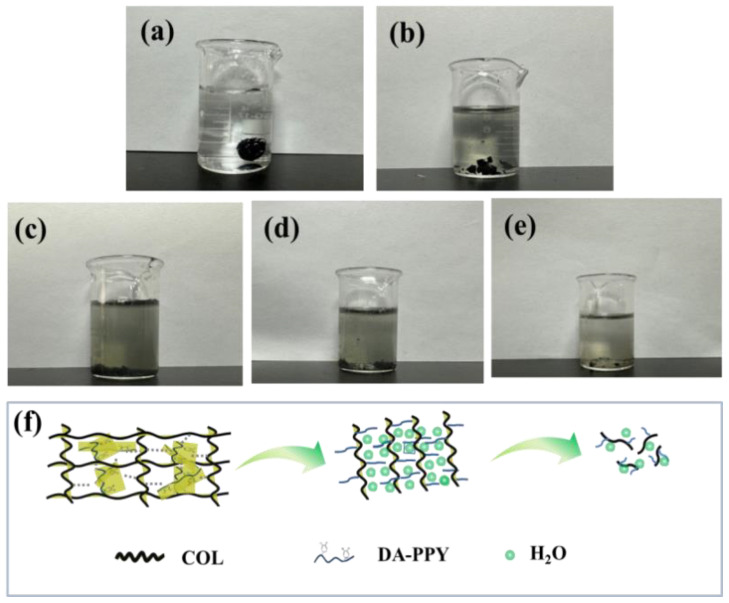
Degradation process of DA-PPY-COL hydrogel soaked in PBS solution of pH 7.4 in the presence of 1% collagenase ((**a**) 1st day, (**b**) 2nd day, (**c**) 5th day, (**d**) 8th day, (**e**) 14th day), and potential degradable mechanism (**f**).

Hydrogel electronic devices with biodegradable properties can reduce the environmental pollution problem caused by electronic product waste and also show potential applications in implantable medical device applications. Figure 5 shows the degradation process of the DA-PPY-COL hydrogels in a PBS solution of pH 7.4 containing 1% collagenase. The hydrogel gradually degraded from a large blocky form into small blocky and granules. After soaking in 1% collagenase, the structure of hydrogel collapsed and became brittle, disintegrating into brittle blocks. Subsequently, the brittle blocks gradually became granules, and the mass of hydrogel became smaller and smaller with a long duration in the alkaline solution, which is due to the water-uptake properties of the hydrogel. A large number of water molecules can penetrate into the interior of the hydrogel, acting as a buffer layer to control the rotation and fracture of collagen molecules. Resultantly, collagen becomes easier to dislocation and hydrolysis [35,36].

In addition, collagenase can also interact with the amino groups in the collagen macromolecules and dissociate them into smaller collagen peptides, making the hydrogel structure loosen and the sensor degradation take place. After two weeks of immersion, the mass loss of the hydrogel was as high as 96% (Figure S4), which is due to the high biodegradability of the collagen. It suggests that abandoned sensors do not cause e-waste and are also expected to be used in implantable medical devices. The potential degradable mechanism is schematically illustrated in Figure 5f. DA-PPY-COL hydrogel was degraded into small fragments of proteins by collagenase, and then it continued to be deeply degraded into smaller peptides or amino acid residues that were dissolved into a solution. To further investigate the in vivo degradation performance, the hydrogels were implanted subcutaneously in mice for 21 days. As shown in Figure S5, the hydrogel nearly completely degraded after 21 days, indicating good biodegradation properties, which are expected to be applied to implantable medical devices.

### 2.6. Electroactivity Measurement

**Figure 6:**
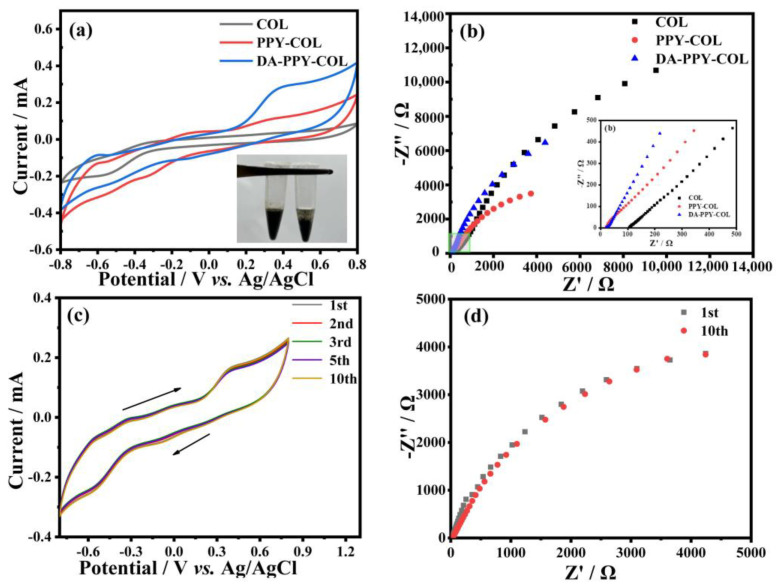
CV plots (**a**) and EIS spectra (**b**) of COL, PPY-COL, and DA-PPY-COL electrodes (insert image of (**a**) is DA-PPY-COL suspension (**left**) and PPY-COL suspension (**right**), insert image of (**b**) is the locally enlarged image of green square), CV curves of DA-PPY-COL electrode after various cycling times at a scan rate of 30 mV s^−1^ (**c**), and EIS spectra of DA-PPY-COL electrode after the 1st and the 10th cycles (**d**).

The electrochemical properties of COL, PPY-COL, and DA-PPY-COL hydrogels were investigated using a three-electrode system. As shown in Figure 6a, the DA-PPY-COL hydrogel demonstrated the largest enclosed area of the cyclic voltammetry (CV) curve. The impedance test results shown in Figure 6b also confirmed that DA-PPY-COL had a lower intrinsic impedance that can be confirmed by the intersection of the impedance spectra and the *X*-axis. In addition, the highest slope of the DA-PPY-COL electrode in the low-frequency region indicates a higher ion diffusion rate in the gel material [37,38], demonstrating the higher electrochemical activity. It is ascribed to the formation of a continuous three-dimensional conductive network mediated by DA in the hydrogel. It can be confirmed by the phenomenon the introduction of DA induces the formation of the homogeneous phase of the pyrrole dispersion system (insert image of Figure 6), promoting the rapid transport of electrons and ions on the electrode surface. Noteworthily, hydrogels containing polypyrrole showed a larger current response than collagen hydrogel due to its π-π conjugated structure. Moreover, the effect of pyrrole doping ratio on the electrochemical performance was investigated through CV and EIS methods. As shown in Figure S6a,b, DA-PPY-COL-8% electrode exhibited the highest enclosed area of the cyclic voltammetry (CV) curve, the lowest intrinsic resistance, and the highest ion diffusion rate in the gel material, implying superior electrochemical activity. A moderate dosage of pyrrole can enhance the ion transport characteristics and electrochemical properties. However, excessive PPY would reduce the ion transport channels and slow down the ion transport rate, similar to the electrochemical behavior of other PPY-based hydrogels reported [4]. Furthermore, the electrochemical properties of hydrogels with ferric chloride and pyrrole in the concentration ratios of 3:1, 4:1, and 5:1 were investigated. Based on CV curves and the area-specific capacitance formula [39], hydrogel electrode prepared with a concentration ratio of 4:1 displayed the highest area-specific capacitance and conductivity, indicating the most superior electrochemical activity, and the corresponding analysis and test results are displayed in Figure S7.

The electrochemical stability of the DA-PPY-COL electrode was further evaluated. As shown in Figure 6c,d, CV profiles and EIS impedance basically did not change greatly after cycling 10 times, indicating that the hydrogel has a more stable electrochemical activity. The conductivity of the hydrogel was tested on the day of synthesis and the fifth day of placement by EIS (Figure S6c). The value of conductivity increases with the increase in the pyrrole content, and as the pyrrole dosage increases to 8%, the conductivity value is the highest, and also higher than the other hydrogels containing PPY. The result is in agreement with the CV determination, implying the conductivity of the DA-PPY-COL hydrogel could be modulated by adjusting the PPY content. Based on the above electrochemical analysis, DA−PPY-COL-8% was selected for subsequent experiments.

### 2.7. Real-Time Motion Detection Using the DA-PPY-COL Hydrogel Strain Sensor

**Figure 7:**
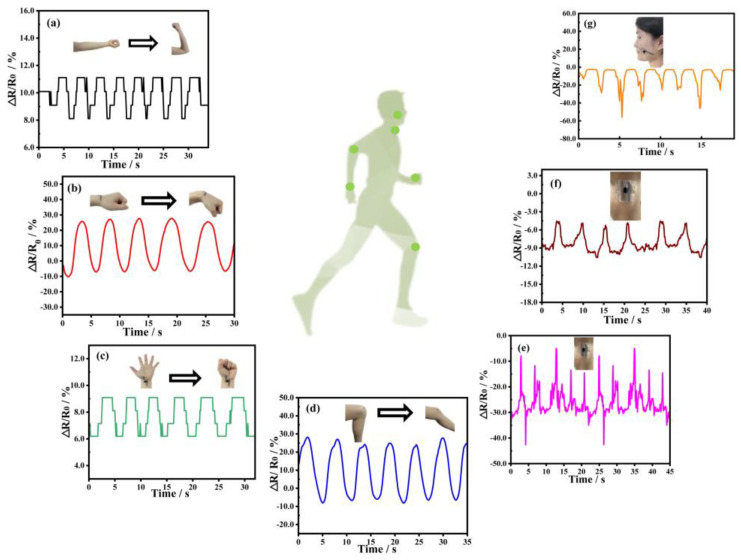
Real-time resistance change curves of the DA-PPY-COL hydrogel sensor for monitoring various human movements including elbow bending (**a**), wrist bending (**b**), fist clenching (**c**), knee bending (**d**), speaking (**e**), swallowing (**f**), and smiling (**g**).

Owing to its good self-adhesive capability, superior biocompatibility, and antibacterial property, DA-PPY-COL hydrogel can firmly adhere to human skin, allowing for the real-time signal exchange of human–machine interfaces. Under the action of external forces, the hydrogel was deformed, and its impedance changed over time, resulting in a change in current in the circuit. As shown in Figure 7a–d, the DA-PPY-COL hydrogel sensor generated a sensitive signal response to large movements, such as elbow bending, wrist bending, fist clenching, and knee bending. When the same action was repeated, the signal peak value of the sensor output was stable and remarkably consistent, indicating excellent real-time stability. Similarly, the hydrogel sensor was also capable of reacting effectively to minute human motions like speaking, swallowing, and smiling (Figure 7e–g). Subsequently, the hydrogel was adhered to the volunteer’s Adam’s apple, which was followed by repeatedly saying “wuhan qinggong daxue”. A steady and almost identical electrical signal was recorded in Figure 7e. Even slight physiological strains induced by tiny muscle movements, such as smiling and swallowing, could also be captured in Figure 7f,g, suggesting that the hydrogel sensor might monitor more than just small movements.

## 3. Materials and Methods

### 3.1. Raw Materials

Bovine Achilles tendon collagen (Molecular Weight 300 KDa) was provided by Collatech Co., Ltd. (Langfang, China). Pyrrole, ferric chloride, and (1-ethyl-(3-dimethy-laminopropyl)) carbodiimide salt (EDC) were purchased from Macklin Biochemical Technology Co., Ltd. (Shanghai, China). Sodium chloride, sodium dihydrogen phosphate, and disodium hydrogen phosphate were purchased from Sinopharm Chemical Reagent Co., Ltd. (Shanghai, China). N-Hydroxysuccinimide (NHS) and dopamine hydrochloride were procured by InnoChem Science & Technology Co., Ltd. (Beijing, China). Calcein-AM and PI (propidium iodide) were procured by Beyotime Biotechnology Inc. (Shanghai, China). *E. coli* and *S. aureus* were provided by Shanghai Ruichu Biotechnology Co., Ltd. (Shanghai, China). Human umbilical vein endothelial cell (HUVEC) was from Tongji Medical College of HUST. (Wuhan, China).

### 3.2. Preparation of DA-PPY-COL Hydrogels

Bovine Achilles tendon collagen (10 mg mL^−1^) was dissolved in a water bath at 37 °C overnight. The above collagen solution with a volume of 4 mL was combined with varying quantities of pyrrole monomer solution, in which the pyrrole volume fraction was 4%, 6%, 8%, and 10%. Subsequently, 94.5 mg of dopamine hydrochloride powder was dispersed into the above mixture with constant stirring. Following thorough stirring, a solution of FeCl_3_ with a molar ratio of 4:1 to pyrrole, as an oxidizing agent, was added dropwise to the aforementioned solution to oxidize the pyrrole with constant stirring for 0.5 h at 4 °C. The obtained emulsion was then transferred to a small Petri dish with a diameter of 3.5 cm and soaked in 100 mL of PBS solution with a pH of 7.4 for 0.5 h to facilitate the formation of an emulsion gel. Subsequently, the gel was crosslinked under 100 mL solution of EDC/NHS in a molar ratio of 3:1 for an overnight soaking period to strengthen the gel network. Ultimately, DA-PPY-COL hydrogel was obtained through a process of repeated soaking and washing with deionized water. Hydrogels containing 4%, 6%, 8%, and 10% pyrrole were named as DA-PPY-COL-4%, DA-PPY-COL-6%, DA-PPY-COL-8%, and DA-PPY-COL-10%, respectively. PPY-COL and COL (pure collagen) hydrogels were prepared for comparison. PPY-COL hydrogel was obtained in the same way as above except that dopamine was not added. Meanwhile, COL hydrogel was prepared as follows. Bovine Achilles tendon collagen with a mass of 100 mg was dissolved in a 10 mL distilled water at 37 °C water bath to form a 10 mg mL^−1^ collagen solution. After dialysis with PBS solution (pH = 7.4) for 1 day, the above collagen solution was incubated at 37 °C for 30 min, and self-assembly collagen hydrogel was formed.

### 3.3. Materials Characterization

Characterization details of the scanning electron microscope (SEM), Fourier transform infrared spectroscopy (FTIR), adhesive property, and rheological analysis of the hydrogel were elaborated in the Supporting Information.

### 3.4. Antibacterial Activity Evaluation

Antibacterial performance was investigated through the bacteriostatic zone method. The hydrogel was delimited into around 1 cm diameter circles and placed in *E. coli* and *S. aureus*. After incubation at 37 °C for 24 h, the diameter of the inhibition zone was measured. Furthermore, the antibacterial activity of the hydrogels was evaluated by the spread plate method. Firstly, the hydrogel (300.0 mg) and *E. coli* or *S. aureus* suspension (100 CFU mL^−1^, 20.0 μL) were added into sterile test tubes. After incubation at 37 °C with a relative humidity of not less than 90% for 2 h and 70 rpm for 24 h, 980 μL of sterilized Luria–Bertani (LB) liquid medium was then added to each sterile tube to re-suspend the stock bacteria. Secondly, the LB agar plate was equally coated with 100 μL of bacterial solution followed by undergoing a 24 h incubation period at 37 °C. Bacteria that had not been treated with hydrogel were generated and set as the control group. Each group contained three parallel samples, the number of bacterial colonies was recorded, and the mean value and standard deviation were calculated from sample data.

### 3.5. Cytotoxicity Evaluation

The cytotoxicity of the hydrogel was evaluated by in vitro cellular experiments. The samples were pretreated before the experiment: the hydrogels were soaked in 75% alcohol for 12 h to remove unreacted monomers and oxidants, then soaked in the bovine serum-free medium solution for 12 h, washed several times, and finally soaked in medium containing 1% double antibody for 3 h for purification, and then placed in a well plate at the end of the sterilization process for use. HUVEC were used for the experiment.

#### 3.5.1. Cell Dead-Viable Staining

The viability of cells was qualitatively evaluated using a live/dead fluorescent staining assay. The cell suspension of 100 μL with a concentration of 5 × 10^4^ cells mL^−1^ was seeded onto a 96-well plate. After incubating for 24 h with hydrogels, the sample was washed three times with 100 μL PBS per well, and the mixed cell staining solution of AM and PI was added and incubated at 37 °C for 60 min. Finally, the plate was gently washed three times using 100 μL PBS per well so as not to be affected by excessive dye and interfere with the observation. An inverted fluorescence microscope (TI2-N-ND-I, Nikon, Tokyo, Japan) was utilized to record images of the cells’ morphology and distribution.

#### 3.5.2. CCK-8 Cell Proliferation Experiment

The proliferation of cells on the extract solution of conducting hydrogel was quantitatively analyzed with CCK-8 kit. The specific process was shown as follows: Firstly, 100 μL cell suspension (5 × 10^4^ cells mL^−1^) was placed on the 96-well plate containing DMEM medium. After the cells were completely attached to the wall, the supernatant was sucked away. Then, 100 μL of DA-PPY-COL-8% hydrogel extract with a concentration of 0.1 mg mL^−1^ was added to the cell culture plate for incubating for 24 h and 48 h, respectively. Subsequently, they were transferred into a constant temperature incubator at 37 °C under a 5% CO_2_ atmosphere for 3 h after adding 10 μL of CCK-8 solution. The blank plate without hydrogel extract and HUVEC was the blank group. The one without the hydrogel extract was the control group. Finally, the cell viability was measured by enzymoleter at 450 nm based on the following Equation (1). Each group of samples contained three parallel samples, and the mean value and standard deviation were calculated. The cell viability was obtained based on Equation (1).

(1) $$\mathrm{Cell}\;\mathrm{viability}=\frac{A_{s}-A_{b}}{A_{c}-A_{b}}\times 100\%$$

Herein, *A*_s_, *A*_c_, and *A*_b_ are the absorbances of the experimental group, control group, and blank group, respectively.

### 3.6. Degradability Test

For the degradability test, the hydrogel sample was cut into the cylinder and immersed in PBS solution of pH 7.4 in the presence of 1% collagenase (125 CDU mg^−1^). After soaking for a period of time, the sample was dried and reweighed, and the degradation ratio was calculated according to Equation (2).

(2) $$\mathrm{Degradation}\;\mathrm{ratio}=\frac{m_{b}-m_{a}}{m_{b}}\times 100\%$$

where *m*_b_ and *m*_a_ represent the mass of hydrogels before and after soaking.

Furthermore, the in vivo degradation experiment was performed by implanting DA-PPY-COL hydrogel in mice. After being embedded for 21 days, the sutures were removed, and the remnants of hydrogel were visually inspected.

### 3.7. Electroactivity Measurement

The electrochemical performance of hydrogels was assessed by cyclic voltammetry (CV) and electrochemical impedance spectroscopy (EIS) techniques on an electrochemical workstation (CHI660E, Shanghai Chenhua, Shanghai, China). The experiment was conducted using a three-electrode setup, which was composed of pure platinum as the counter electrode, Ag/AgCl electrode as the reference electrode, hydrogel attached to the conductive glass (ITO) substrate as the working electrode, and PBS solution of 0.1 M (pH = 7.4) as an electrolyte. A CV determination for the hydrogel was performed in the potential range between −0.8 and 0.5 V at a sweep rate of 50 mV s^−1^. EIS was recorded at frequencies from 0.1 Hz to 10 kHz with an amplitude of ±5 mV.

The conductivity of the hydrogel was obtained by attaching two pieces of copper foil onto the cross-section of hydrogel using EIS techniques, and the determination was repeated five times. The corresponding conductivity (σ) was calculated by the following equation.

(3) $$\sigma =\frac{L}{RS}$$

Herein, *R* and *L* are the resistance and the length of the hydrogel, respectively, and *S* is the contact area of the hydrogel with the copper sheet electrodes.

### 3.8. Sensing Capability of the Hydrogels

To demonstrate the strain test, the hydrogels were attached to various parts of the volunteers to monitor the resistance variations in hydrogels during large-scale movements and tiny motions. The corresponding signals were recorded in real-time by the electrochemical workstation (CH Instruments Model 600E, Shanghai, China). The variation rate of the resistance was calculated based on Equation (4):

(4) $$\frac{\Delta R}{R}=\frac{R-R_{0}}{R_{0}}\times 100\%$$

where *R*_0_ and *R* represent the raw resistance and real-time resistance of the hydrogel, respectively.

### 3.9. Statistical Analysis

Data were gathered in triplicate and presented as mean ± standard deviation using SPSS 22.0 software. The level of significance was assessed in LSD post hoc tests in conjunction with a one-way analysis of variance (ANOVA). In all statistical evaluations, a probability (*p*) value of less than 0.001 (***) was deemed highly significant.

## 4. Conclusions

In this work, we proposed a method to construct PDA-PPY-COL hydrogel using DA-mediated pyrrole in situ polymerization within the collagen. The hydrogels, with excellent self-adhesion, flexibility, biodegradability, electroactivity, and antimicrobial properties, can act as a stress sensor to stably and sensitively detect human motion movements and fine micro-expressions, which is ascribed to the formation of the homogeneous phase of the pyrrole dispersion system induced by DA, promoting the rapid transport of electrons and ions on the electrode surface. This work provides a prospect for developing multifunctional CHs applied in motion sensing, biomimetic electronic skin, and implantable medical devices. Despite its superior biocompatibility and electrochemical properties, PDA-PPY-COL hydrogel suffers from several drawbacks, such as poor mechanical properties, lack of self-healing capability, unsatisfied water retention capacity, and the absence of anti-freezing capability. Addressing the above problems is essential to advancing its practical applications.

## Data Availability

The raw data supporting the conclusions of this article will be made available by the authors on request.

## Supplementary Materials

The following supporting information can be downloaded at: https://www.mdpi.com/article/10.3390/molecules29235728/s1, Figure S1: Dynamic rheological behaviors of COL and DA-PPY-COL hydrogels with different PPY contents; Figure S2: Fluorescence micrographs of HUVEC (living (a1, b1, c1, and d1), dead (a2, b2, c2, and d2) and merged (a3, b3, c3, and d3)) cultivated in the culture media without hydrogel extract (a1,a2, and a3), and containing COL (b1, b2, and b3), PPY-COL (c1, c2, and c3), and DA-PPY-COL (d1, d2, and d3) hydrogel extract. All the data are expressed as means ± SD (standard deviation, *n* = 3); Figure S3: Cell viability of HUVEC in control group and the experimental group containg PPY-COL and DA-PPY-COL hydrogel extract. Figure S4: Plot of degradation rate over time. Figure S5: The picture of in vivo degradability in mice for 0 day (a) and 21 days (b). Figure S6: CV plots (a), EIS spectra (b) of COL, DA-PPY-COL-4%, DA-PPY-COL-6%, DA-PPY-COL-8%, and DA-PPY-COL-10% electrodes (inset image: local enlarged EIS spectra), and conductivity of COL, DA-PPY-COL-4%, DA-PPY-COL-6%, DA-PPY-COL-8%, and DA-PPY-COL-10% hydrogels (c). Figure S7: CV plots (a), capacities (b), EIS spectra (c, inset image is local enlarged EIS spectra), and conductivities (d) of DA-PPY-COL-8% hydrogel electrodes prepared with FeCl_3_ and PY in the concentration ratios of 3:1, 4:1, and 5:1.

## References

[1] Duan Z., Jiang Y., Huang Q., Yuan Z., Zhao Q., Wang S., Zhang Y., Tai H. (2021). A do-it-yourself approach to achieving a flexible pressure sensor using daily use materials. J. Mater. Chem. C.

[2] Zhang Z., Li Q., Xu L., Tian W., Li Z. (2024). High-performance flexible temperature sensors based on laser-irradiated Ag-MWCNTs/PEDOT: PSS. ACS Appl. Mater. Interfaces.

[3] Han Y., Wei H., Du Y., Li Z., Feng S., Huang B., Xu D. (2023). Ultrasensitive flexible thermal sensor arrays based on high-thermopower ionic thermoelectric hydrogel. Adv. Sci..

[4] Yang J., Gao M., Wang K., Yu H., Lv R., Fu M. (2023). Robust double-network polyvinyl alcohol-polypyrrole hydrogels as high-performance electrodes for flexible supercapacitors. J. Colloid Interface Sci..

[5] Tao X., Ye S., Zhu K., Dou L., Cui P., Ma J., Zhao C., Wei X., Guo L., Hojjati-Najafabadi A. (2023). ATMP doped conductive PANI/CNTs composite hydrogel electrodes toward high energy density flexible supercapacitors. ACS Appl. Energy Mater..

[6] Ling Q., Fan X., Ling M., Liu J., Zhao L., Gu H. (2023). Collagen-based organohydrogel strain sensor with self-healing and adhesive properties for detecting human motion. ACS Appl. Mater. Interfaces.

[7] Wang T., Yi W., Zhang Y., Wu H., Fan H., Zhao J., Wang S. (2023). Sodium alginate hydrogel containing platelet-rich plasma for wound healing. Colloid Surf. B.

[8] Liu F., Wang L., Zhai X., Ji S., Ye J., Zhu Z., Teng C., Dong W., Wei W. (2023). A multi-functional double cross-linked chitosan hydrogel with tunable mechanical and antibacterial properties for skin wound dressing. Carbohyd. Polym..

[9] Aycan D., Karaca F., Alemdar N. (2023). Development of hyaluronic acid-based electroconductive hydrogel as a sensitive non-enzymatic glucose sensor. Mater. Today Commun..

[10] Chen M., Wan H., Hu Y., Zhao F., An X., Lu A. (2023). Rationally designed cellulose hydrogel for an ultrasensitive pressure sensor. Mater. Horiz..

[11] Li X., Zhang S., Li X., Lu L., Cui B., Yuan C., Guo L., Yu B., Chai Q. (2023). Starch/polyvinyl alcohol with ionic liquid/graphene oxide enabled highly tough, conductive and freezing-resistance hydrogels for multimodal wearable sensors. Carbohyd. Polym..

[12] Roshanbinfar K., Kolesnik-Gray M., Angeloni M., Schruefer S., Fiedler M., Schubert D., Ferrazzi F., Krstic V., Engel F. (2023). Collagen hydrogel containing polyethylenimine-gold nanoparticles for drug release and enhanced beating properties of engineered cardiac tissues. Adv. Healthc. Mater..

[13] He Z., Shen J., Lan M., Gu H. (2024). Collagen fiber-reinforced, tough and adaptive conductive organohydrogel e-skin for multimodal sensing applications. J. Mater. Chem. B.

[14] Zhao R., Luo J., Ke T., Zhang J., Astruc D., Zhou J., Gu H. (2024). Ultra-Tough, highly stable and Self-Adhesive Goatskin-Based intelligent Multi-Functional organogel e-skin as Temperature, Humidity, Strain, and bioelectric four-mode sensors for health monitoring. Chem. Eng. J..

[15] Liu X., Cui B., Wang X., Zheng M., Bai Z., Yue O., Fei Y., Jiang H. (2023). Nature-skin-derived e-skin as versatile “wound therapy-health monitoring” bioelectronic skin-scaffolds: Skin to bio-e-skin. Adv. Healthc. Mater..

[16] Zhu L., Yu Z., Li S., Xu C., Hou Y., Liao L., Xu Y., Zhang J., Wei B., Wen W. (2024). Recent advances on collagen biomaterial: From extraction, cross-linking to tissue regeneration. Polym. Rev..

[17] Han Q., Koyama T., Watabe S., Nagashima Y., Ishizaki S. (2023). Isolation and characterization of collagen and collagen peptides with hyaluronidase inhibition activity derived from the skin of marlin (Istiophoridae). Molecules.

[18] Wei B., Huang S., Li K., Wu H., Liu Y., Zhang J., Hou Y., Zhu L., Xu C., Wang L. (2024). Recognition of MCF-7 breast cancer cells using native collagen probes: Collagen source effect. Int. J. Biol. Macromol..

[19] Cai P., Zheng B., Xu Y., Li B., Liu Z., Huang Y., Ye J., Xiao M. (2024). Multifunctional fish-skin collagen-based hydrogel sealant with dual-dynamic-bond cross-linked for rapid hemostasis and accelerated wound healing. Int. J. Biol. Macromol..

[20] Song B., Fan X., Shen J., Gu H. (2023). Ultra-stable and self-healing coordinated collagen-based multifunctional double-network organohydrogel e-skin for multimodal sensing monitoring of strain-resistance, bioelectrode, and self-powered triboelectric nanogenerator. Chem. Eng. J..

[21] Gao C., Zheng D., Long B., Chen Z., Zhu J., Gao Q. (2023). Anti-swelling and adhesive γ-PGA/PVA/PEDOT: PSS/TA composite conductive hydrogels for underwater wearable sensors. Eur. Polym. J..

[22] Paradee N., Thanokiang J., Sirivat A. (2021). Conductive poly (2-ethylaniline) dextran-based hydrogels for electrically controlled diclofenac release. Mat. Sci. Eng. C-Mater..

[23] Taha G.M., Baseer R.A., Kassem A.F., Khalil R. (2022). Designing of semiconductive cotton fabrics based on poly (propynyl benzo thiazolone) with UV protection and antibacterial properties. Mat. Sci. Eng. B-Adv..

[24] Nada A., Eckstein Andicsová A., Mosnáček J. (2022). Irreversible and self-healing electrically conductive hydrogels made of bio-based polymers. Int. J. Mol. Sci..

[25] Rahmani P., Shojaei A., Dickey M. (2024). A highly conductive and ultra-stretchable polyaniline/cellulose nanocrystal/polyacrylamide hydrogel with hydrophobic associations for wearable strain sensors. J. Mater. Chem. A.

[26] Hu S., Zhou L., Tu L., Dai C., Fan L., Zhang K., Yao T., Chen J.Q., Wang Z., Xing J. (2019). Elastomeric conductive hybrid hydrogels with continuous conductive networks. J. Mater. Chem. B.

[27] Liu X., Shi H., Song F., Yang W., Yang B., Ding D., Liu Z., Hui L., Zhang F. (2024). A highly sensitive and anti-freezing conductive strain sensor based on polypyrrole/cellulose nanofiber crosslinked polyvinyl alcohol hydrogel for human motion detection. Int. J. Biol. Macromol..

[28] Zhang L., Wu Y., Xia Y., Jin L. (2022). High capacitance of polypyrrole hydrogel electrode synthesized by polymerization of conjugated pyrrole salt. Electrochim. Acta.

[29] He Y., Li Z., Su H., Sun Y., Shi W., Yi Y., Ge D., Fan Z. (2023). Pyrrole-doped polydopamine-pyrrole (pda-npy) nanoparticles with tunable size and improved nir absorption for photothermal therapy. Pharmaceuticals.

[30] Lei H., Zhao J., Ma X., Li H., Fan D. (2021). Antibacterial dual network hydrogels for sensing and human health monitoring. Adv. Healthc. Mater..

[31] Lv Y., Chen C., Jin L., Zheng Y., Wu S., Zhang Y., Li Z., Zhu S., Jiang H., Cui Z. (2023). Microwave-excited, antibacterial core-shell BaSO_4_/BaTi_5_O_11_@PPy heterostructures for rapid treatment of *S. aureus*-infected osteomyelitis. Acta Biomater..

[32] Maruthapandi M., Saravanan A., Gupta A., Luong J., Gedanken A. (2022). Antimicrobial activities of conducting polymers and their composites. Macromol.

[33] Borges M., Nagay B., Costa R., Souza J., Mathew M., Barão V. (2023). Recent advances of polypyrrole conducting polymer film for biomedical application: Toward a viable platform for cell-microbial interactions. Adv. Colloid Interface Sci..

[34] Ding X., Duan S., Ding X., Liu R., Xu F. (2018). Versatile antibacterial materials: An emerging arsenal for combatting bacterial pathogens. Adv. Funct. Mater..

[35] Zhang W., Pan Z., Ma J., Wei L., Chen Z., Wang J. (2022). Degradable cross-linked collagen fiber/MXene composite aerogels as a high-performing sensitive pressure sensor. ACS Sustain. Chem. Eng..

[36] Guo B., Zhong Y., Chen X., Yu S., Bai J. (2023). 3D printing of electrically conductive and degradable hydrogel for epidermal strain sensor. Compos. Commun..

[37] Li W., Yang Y., Zhang X., Liu Y., Luan J. (2024). Fabrication and assembly of supercapacitors based on Ni-based MOFs and their derivative materials for enhancing their electrochemical performances. Nanoscale.

[38] Li L., Ai Z., Wu J., Liu Z., Huang M., GAO Y., Bai H. (2024). A robust polyaniline hydrogel electrode enables superior rate capability at ultrahigh mass loadings. Nat. Commun..

[39] Ge J., Zhu M., Eisner E., Yin Y., Dong H., Karnaushenko D., Karnaushenko D.D., Zhu F., Ma L., Schmidt O. (2021). Imperceptible supercapacitors with high area-specific capacitance. Small.

