# Supplementary material for: Adhesive, Biocompatible, Antibacterial, and Degradable Collagen-Based Conductive Hydrogel as Strain Sensor for Human Motion Monitoring
Source: Molecules. 2024 Dec 4;29(23):5728. doi: 10.3390/molecules29235728 (PMC11643890; doi:10.3390/molecules29235728)
Supplement: Supplementary file 1 [file molecules-29-05728-s001.zip › molecules-3326069-supplementary-revised after pub.pdf]

Experimental section

Scanning electron microscope (SEM)

The morphology of hydrogel was observed by field emission scanning electron microscopy(SEM, Regulus8100, Hitachi, Japan) at an accelerated voltage of 10 KV. Before test, the lyophilized hydrogel samples were placed in liquid nitrogen for about 20 s. Immediately after removal, the samples were brittlely broken by tweezers, stuck to the sample stage with the cross-section facing upwards, and sprayed with gold for 60 s.

Fourier transform infrared spectroscopy (FTIR)

The chemical structure of hydrogel was analyzed by Fourier transform infrared (FTIR) spectrometer (Nicolet is10, Thermo Fisher Scientific, Tokyo, USA). According to the requirements of the infrared compression method, a small amount of lyophilized hydrogel powder and potassium bromide crystals (mass ratio about 1:50 000) were ground into a powder and then pressed into a transparent sheet for FTIR testing.

Rheological analysis

The rheological properties of the hydrogels were tested by rotational rheometer, where the elastic modulus (G') and loss modulus (G'') of the hydrogels were measured at a constant strain amplitude of 2.0% and a frequency interval of 0.1-10 Hz, and the change in elastic modulus of the samples was statistically analyzed at 10 Hz. A PP-40 (40 mm) parallel plate detector was used in the assay.

Adhesive property

Adhesion is a significant property of hydrogel strain sensors in practical applications. The adhesion characteristics of DA-PPY-COL hydrogel were vividly demonstrated by adhering to metal, wood, rubber, glass, plastic, and skin.

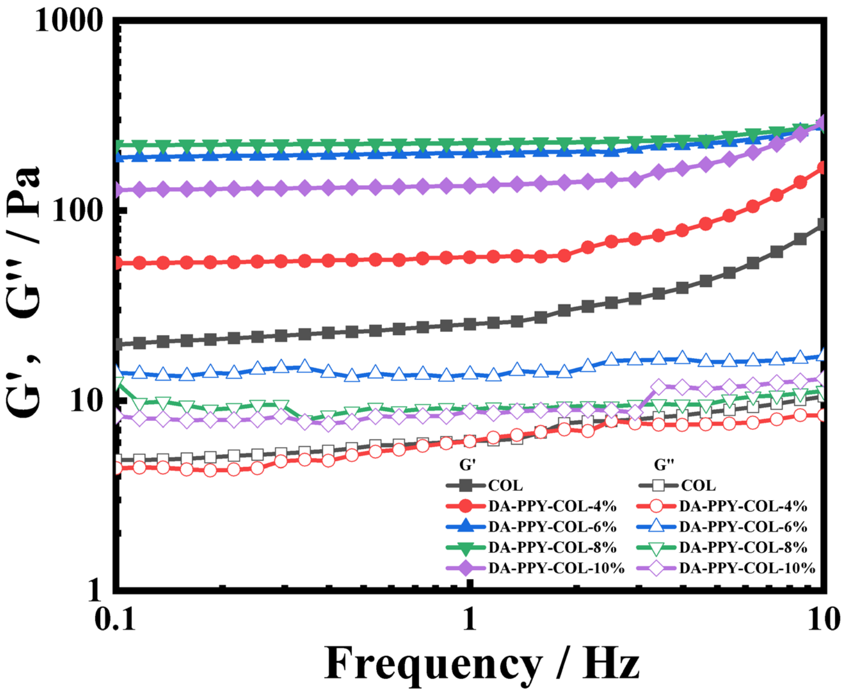

Figure S1 Dynamic rheological behaviors of COL and DA-PPY-COL hydrogels with different PPY contents.

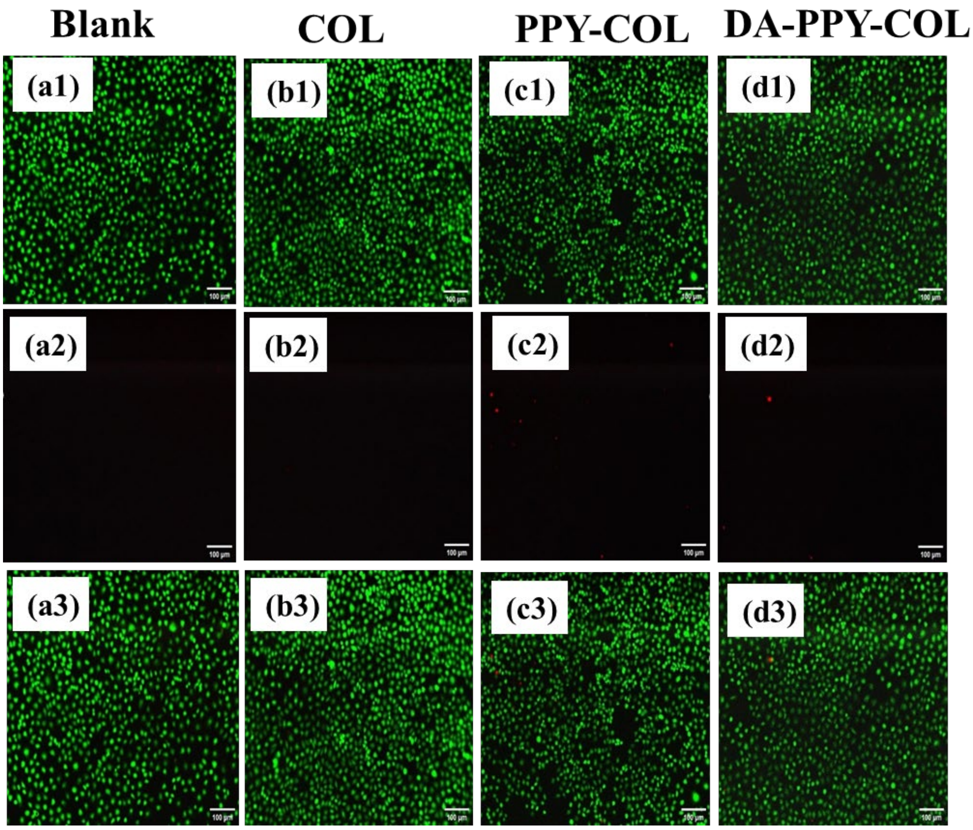

Figure S2 Fluorescence micrographs of HUVEC cells (living (a1, b1, c1, and d1), dead (a2, b2, c2, and d2)) and merged (a3, b3, c3, and d3)) cultivated in the culture media without hydrogel extract (a1,a2, and a3), and containing COL (b1, b2, and b3), PPY-COL (c1, c2, and c3), and DA-PPY-COL (d1, d2, and d3) hydrogel extract. All the data are expressed as means ± SD (standard deviation, n = 3).

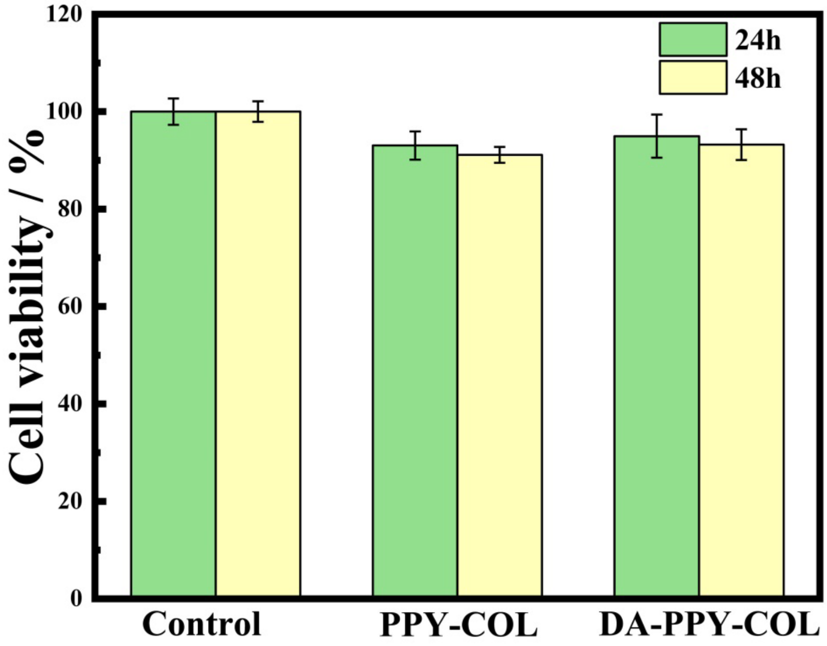

Figure S3 Cell viability of HUVEC cells in control group and the experimental group containg PPY-COL and DA-PPY-COL hydrogel extract.

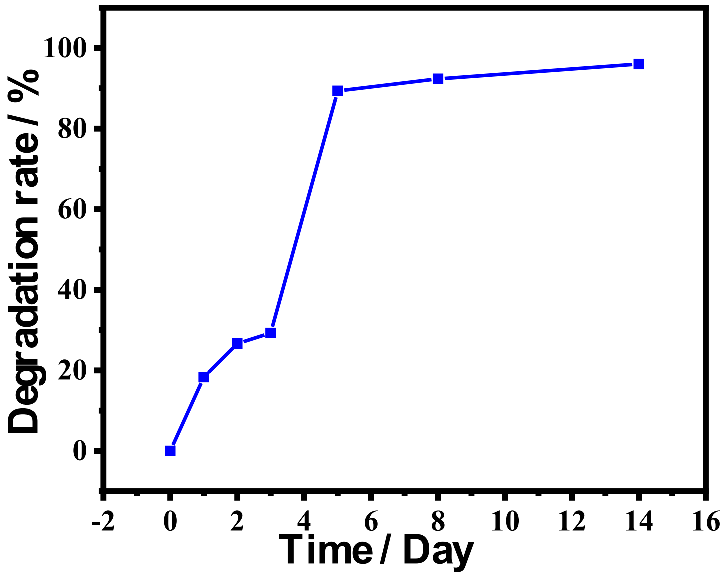

Figure S4 Plot of degradation rate over time.

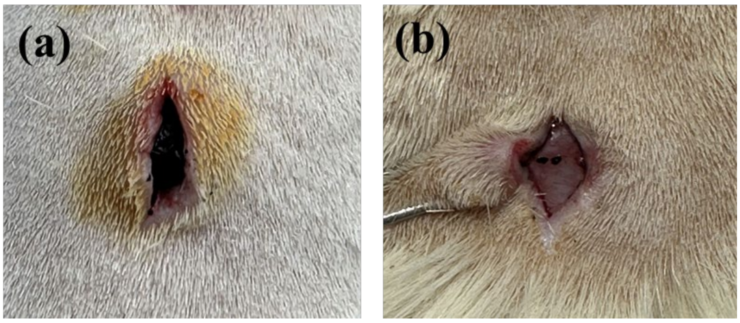

Figure S5 The picture of in vivo degradability in mice for 0 day (a) and 21 days (b).

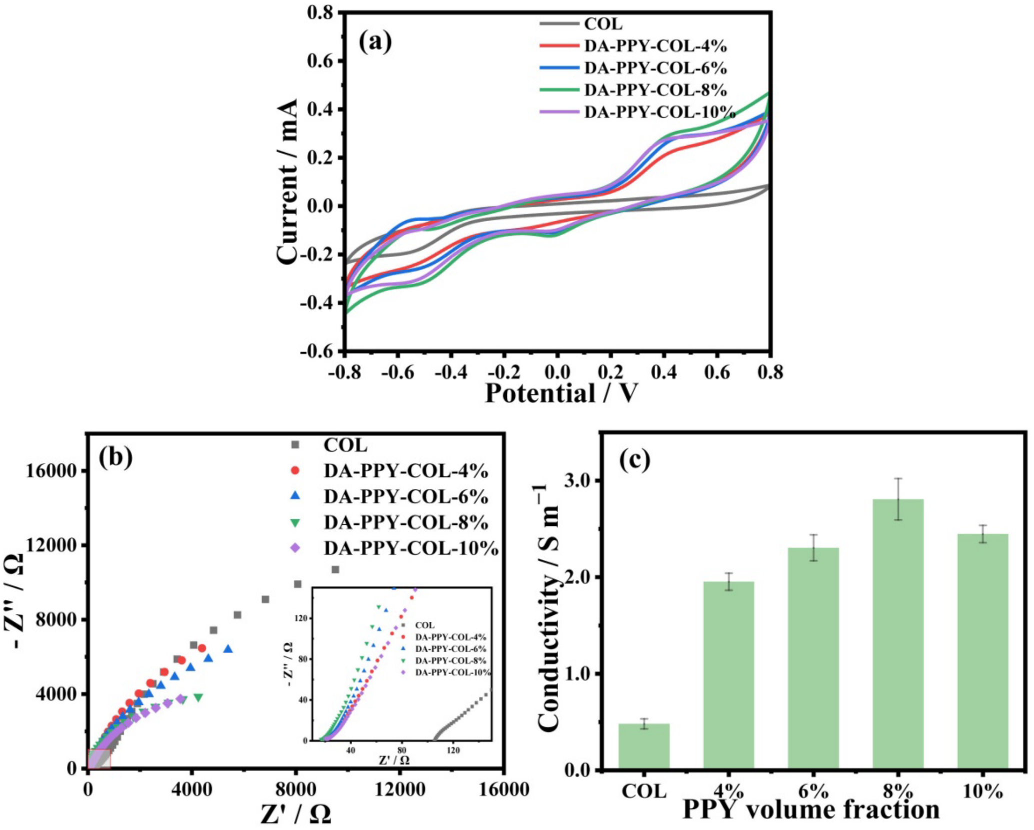

Figure S6 CV plots (a), EIS spectra (b) of COL, DA-PPY-COL-4%, DA-PPY-COL-6%, DA-PPY-COL-8%, and DA-PPY-COL-10% electrodes (inset image: local enlarged EIS spectra), and conductivity of COL, DA-PPY-COL-4%, DA-PPY-COL-6%, DA-PPY-COL-8%, and DA-PPY-COL-10% hydrogels (c).

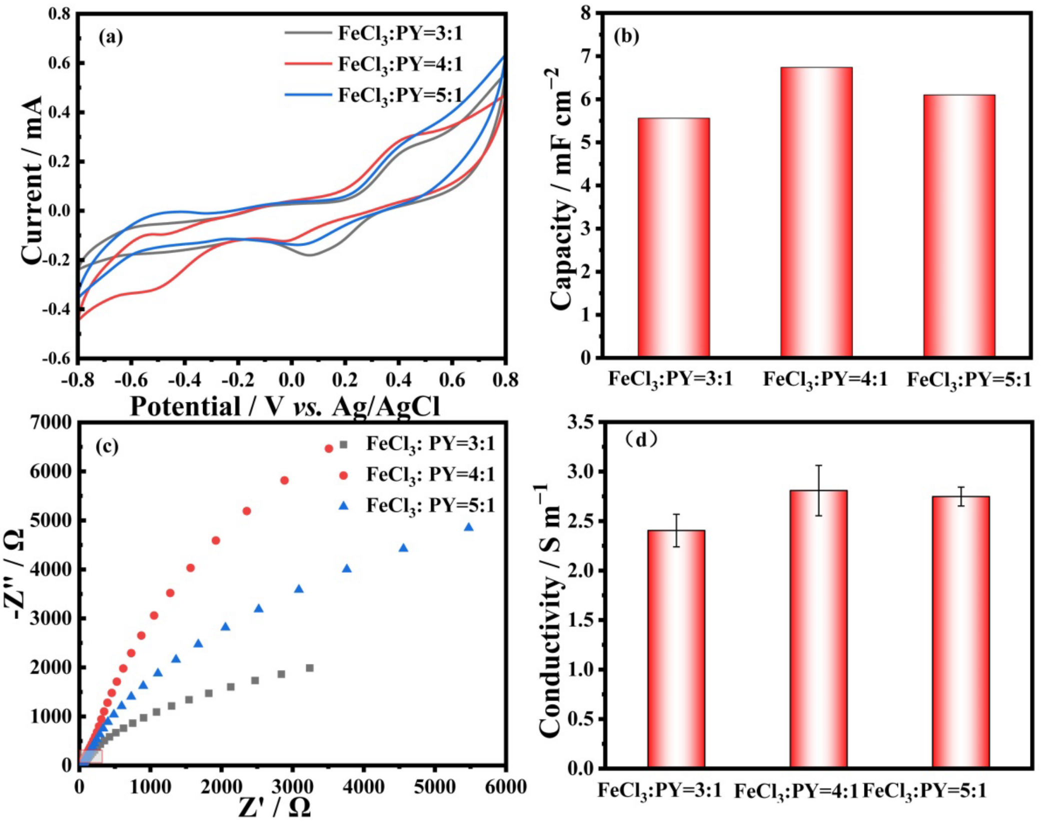

Figure S7 CV plots (a), capacities (b), EIS spectra (c, inset image is local enlarged EIS spectra), and conductivities (d) of DA-PPY-COL-8% hydrogel electrodes prepared with FeCl3 and PY in the concentration ratios of 3:1, 4:1, and 5:1.

The area-specific capacitances based on CV curves were calculated on the following formula [1]:

$$C_{\mathrm{cv}} = \frac{\int_{V_{\mathrm{i}}}^{V_{\mathrm{f}}} I \mathrm{d}V}{\nu A(V_{\mathrm{f}} - V_{\mathrm{i}})} \qquad \text{(S1)}$$

Where C (F cm^{-2}) is the area-specific capacitance based on the coating area of the active materials on the working electrode surface, I (A) is the discharge current, Δt (s) is the time consumed in potential window of ΔV (V) during the discharge process, A (cm^2) is coating area of the active materials, V_{i} is the initial potential, V_{f} is the final potential (V), and ν (V/s) is the scan rate.

[1] Ge, J.; Zhu, M.; Eisner, E.; Yin, Y.; Dong, H.; Karnaushenko, D.; Karnaushenko, D. D.; Zhu, F.; Ma, L.; Schmidt, O. Imperceptible supercapacitors with high area-specific capacitance. Small 2021, 17(24), 2101704.
